# Prevalence and Determinants of Sex-Specific Dietary Supplement Use in a Greek Cohort

**DOI:** 10.3390/nu13082857

**Published:** 2021-08-20

**Authors:** Marina O. Rontogianni, Afroditi Kanellopoulou, Georgios Markozannes, Emmanouil Bouras, Christos Derdemezis, Michail T. Doumas, Dimitrios E. Sigounas, Vasilios T. Tzovaras, Konstantinos Vakalis, Demosthenes B. Panagiotakos, Eleni Aretouli, Ioanna Tzoulaki, Evangelos Evangelou, Evangelos C. Rizos, Evangelia Ntzani, Konstantinos K. Tsilidis

**Affiliations:** 1 Department of Hygiene and Epidemiology, University of Ioannina School of Medicine, 45110 Ioannina, Greece; marinaront@gmail.com (M.O.R.); afkanellopoulou@gmail.com (A.K.); georgemarkozannes@gmail.com (G.M.); ebouras@auth.gr (E.B.); cderdemezis@yahoo.gr (C.D.); jtzoulaki@gmail.com (I.T.); vangelis@uoi.gr (E.E.); entzani@uoi.gr (E.N.); 2 Department of Hygiene, Social-Preventive Medicine and Medical Statistics, Aristotle University of Thessaloniki School of Medicine, 54124 Thessaloniki, Greece; 3 Ioannina Medical Care, 45333 Ioannina, Greece; michalis_doumas@yahoo.gr (M.T.D.); dsigounas@gmail.com (D.E.S.); vtzovi@yahoo.gr (V.T.T.); kosvak@yahoo.gr (K.V.); 4 Department of Nutrition and Dietetics, School of Health Science and Education, Harokopio University, 17676 Athens, Greece; dbpanag@hua.gr; 5 Discipline of Nutrition and Dietetics, Faculty of Health, University of Canberra, Canberra 2617, Australia; 6 School of the Social Sciences, University of Ioannina, 45110 Ioannina, Greece; eleni.aretouli@gmail.com; 7 Laboratory of Cognitive Neuroscience, School of Psychology, Aristotle University of Thessaloniki, 54124 Thessaloniki, Greece; 8 Department of Epidemiology and Biostatistics, School of Public Health, Imperial College London, London W2 1PG, UK; 9 Department of Internal Medicine, University Hospital of Ioannina, 45500 Ioannina, Greece; vagrizos@gmail.com; 10 School of Medicine, European University of Cyprus, Nicosia 2404, Cyprus; 11 Center for Evidence-Based Medicine, Department of Health Services, Policy and Practice, School of Public Health, Brown University, Providence, RI 02912, USA; 12 Institute of Biosciences, University Research Center of loannina, University of Ioannina, 45110 Ioannina, Greece

**Keywords:** dietary supplements, prevalence, determinants, Epirus Health Study, Greece

## Abstract

We describe the profile of dietary supplement use and its correlates in the Epirus Health Study cohort, which consists of 1237 adults (60.5% women) residing in urban north-west Greece. The association between dietary supplement use and demographic characteristics, lifestyle behaviors, personal medical history and clinical measurements was assessed using logistic regression models, separately for women and men. The overall prevalence of dietary supplement use was 31.4%, and it was higher in women (37.3%) compared to men (22.4%; *p*-value = 4.2^−08^). Based on multivariable logistic regression models, dietary supplement use in women was associated with age (positively until middle-age and slightly negatively afterwards), the presence of a chronic health condition (OR = 1.71; 95% CI, 1.18–2.46), lost/removed teeth (OR = 0.52; 95% CI, 0.35–0.78) and diastolic blood pressure (OR per 5 mmHg increase =0.84; 95% CI, 0.73–0.96); body mass index and worse general health status were borderline inversely associated. In men, dietary supplement use was positively associated with being employed (OR = 2.53; 95% CI, 1.21–5.29). A considerable proportion of our sample used dietary supplements, and the associated factors differed between women and men.

## 1. Introduction

Dietary supplement (DS) use is a popular diet-related behaviour among adults with an estimated global industry of 140.3 billion USD in 2020 [1]. Several studies highlight an increasing trend of DS use during the last decades [2,3,4]. Variations on the prevalence of use are evident across countries with more than half of the US and Danish [2,5] and more than 30% of the Dutch, Canadian, Australian and Korean adult populations [6,7,8,9,10] using DS. On the contrary, the prevalence estimate for the Chinese population (aged 6 years or older) is 0.71% [11]. National survey data for DS use in the general population of low and middle-income countries remain limited. Demographic characteristics, socioeconomic status and lifestyle factors may act as determinants of DS use, and studies have shown that users were more likely to be women [12], physically active [8,10], non-smokers [7], older [2,13], of higher income [14] and educational level [15].

Up to date data on DS use and its plausible correlates in the Greek population are scarce. According to the results of a single 24-h dietary recall used in the context of the European Prospective Investigation into Cancer and Nutrition (EPIC) calibration study, 2% of men and 6.7% of women aged 35 to 74 years used DS in the 1990s [16]. In a 2004 cross-sectional study that used a convenience sample of 123 young women residents of a medium-sized Greek town, about one in four reported vitamin use at least once a week [17]. Considering the high rates of DS use worldwide and the lack of contemporary data in Greece, the aim of this study was to describe the prevalence of and identify factors associated with DS use among the participants of a Greek cohort study, the Epirus Health Study. Additional and updated information regarding the prevalence and predictors of DS use will aid the formulation of appropriate and effective future interventions for its proper use.

## 2. Materials and Methods

The paper has been written in accordance with the Strengthening the Reporting of Observational Studies in Epidemiology (STROBE) guidelines [18].

### 2.1. Study Design

The present study was based on the Epirus Health Study (EHS) (https://ehs.med.uoi.gr/, (accessed on 14 July 2021)), an ongoing population-based prospective cohort study conducted in the Epirus region in the north-west of Greece since June 2019. All participants were interviewed face-to-face with the use of a standard questionnaire with close-ended questions, and they attended a series of extensive clinical examinations at recruitment, except for 40 participants who joined the study during the mass lockdown period (23 March–18 May 2020), for whom telephone-based interviews were conducted. Detailed description of the study design, aims and recruitment procedures are provided elsewhere [19]. The analytical sample size comprised of 1237 individuals aged between 20 and 80 years and recruited up to 8 March 2021.

### 2.2. Assessment of Exposure

DS are defined as “foodstuffs the purpose of which is to supplement the normal diet and which are concentrated sources of nutrients or other substances with a nutritional or physiological effect, alone or in combination, marketed in dose form, namely forms such as capsules, pastilles, tablets, pills and other similar forms, sachets of powder, ampoules of liquids, drop dispensing bottles, and other similar forms of liquids and powders designed to be taken in measured small unit quantities’’ (Directive 2002/46/EC, European Parliament and Council, 2002) [20]. Participants were asked at recruitment whether they had used any DS during the previous week. In case of a positive answer, the names of the DS were recorded. For verification purposes, participants were reminded to bring their supplement boxes with them at the recruitment visit. Participants were categorized regarding DS use, based on the number of vitamins they contained (no DS use vs. DS containing one vitamin vs. DS containing more than one vitamin vs. DS containing no vitamins). However, since complete information on the content of each DS was available in 45% of the users, and due to high possibility of recall error when DS use was self-reported, we chose to use the dichotomous use vs. no use variable in the statistical analysis.

### 2.3. Other Covariates

All participants provided information on socio-demographic characteristics, general health status, personal and family medical history and lifestyle factors. Clinical examinations at the baseline visit included anthropometry (measured weight, height, waist and hip circumference), bioelectrical impedance analysis, blood pressure monitoring, arterial stiffness, and oximetry. Fasting blood samples were also collected at recruitment, and glucose and lipids were measured in all participants. More information on the captured variables, data collection procedures and quality control are provided elsewhere [19]. A series of variables were assessed a posteriori using participants’ baseline information. We calculated the Mediterranean Diet Adherence Screener (MEDAS) score (range 0 to 14) to estimate the participants’ adherence to Mediterranean diet, with higher scores indicating better adherence [21]. The Pittsburgh Sleep Quality Index (PSQI) was used to assess the participants’ sleep quality. The score ranges from 0 to 21 and the higher the score, the poorer the sleep quality [22]. Participants were classified as non-smokers, former smokers and current smokers according to their self-reported smoking habits. Recreational physical activity was assessed in days per week and minutes per day. We estimated the total metabolic equivalents of energy expenditure (MET) as the sum of activity-specific METs/week by multiplying the hours of activity per week with an activity-specific MET score (3.0 for walking, 6.0 for moderate-intensity and 9.0 for vigorous-intensity physical activity) [23].

### 2.4. Statistical Analysis

Participants’ baseline characteristics were summarized using means and standard deviations (SD) for continuous variables and counts and percentages for categorical variables. Study characteristics were presented overall and according to sex and DS use. Chi-squared and independent samples *t*-test were used to detect any differences between subgroups for categorical and continuous variables, respectively. Using data obtained from the Hellenic Statistical Authority, we estimated the prevalence of DS use after standardizing to the corresponding age and sex distributions of the populations in Greece and Epirus. Due to the observed differences in the prevalence of supplement users between women and men and given that the distribution of many of the potential determinants regarding DS use differed by sex, we investigated potential correlates of DS use separately for women and men. The association between age and DS use was assessed both linearly and non-linearly using restricted cubic splines with five knots in the analysis, due to previous literature findings regarding a potential non-linear association between age and DS use [9,24].

The analysis implemented a two-step selection procedure. In the 1st step, a univariable logistic regression model was fitted for each of the 37 a priori selected factors as potential determinants of DS use. The variables found to be statistically significant at 0.10 in the univariable models were then fitted in a multivariable logistic regression model. A sensitivity analysis was performed using Collett’s four-step model selection procedure [25]. In this procedure, univariable logistic regression models were fitted for each variable (step 1), and those found significant were included in a multivariable model (step 2). The non-significant variables from step 1 were added to this model and re-examined in terms of statistical significance (step 3), and a final analysis was then performed using a stepwise backward approach (step 4). Level of statistical significance for steps 1 to 3 was 0.10, and 0.05 for step 4.

Potential multicollinearity among the independent variables in the final models was examined by calculating the variance inflation factor (VIF) of each variable. The examined determinants of DS use had low percentage of missing values; thus, a complete case analysis was performed.

Statistical analyses were performed using STATA (version 14; StataCorp, College Station, TX, USA).

## 3. Results

The total number of participants was 1237 consisting of 748 women and 489 men. The mean age of our sample was 48.1 (SD = 11) years and was similar by sex (*p*-value = 0.79). The majority of the other examined variables showed different distributions by sex (Table 1). Most of the participants had tertiary education (67%), were employed (75.3%) and reported having good or very good general health status (87.9%). Only 1 in 10 women reported monthly income of more than 1400 euros, compared to 1 in 4 men (*p*-value = 3.1^−15^). Frequent alcohol consumption (more than twice/week) was less common in women than men (9% vs. 21.1%, *p*-value = 1.8^−15^), and women were more likely to be never smokers (37.2 vs. 29.7, *p*-value = 2.6^−03^). The percentage of women self-reporting a personal history of chronic condition was significantly higher than for men (45.4% vs. 34.8%, *p*-value = 2.5^−04^). Additionally, the prevalence of medication use was higher in women than men (39.2% vs. 32.5%, *p*-value = 0.05). Compared to men, women had lower anthropometric (except for hip circumference and % fat), blood pressure and biomarker (except for total and high-density lipoprotein (HDL) cholesterol) measurements (all *p*-values < 0.001).

**Figure 1:**
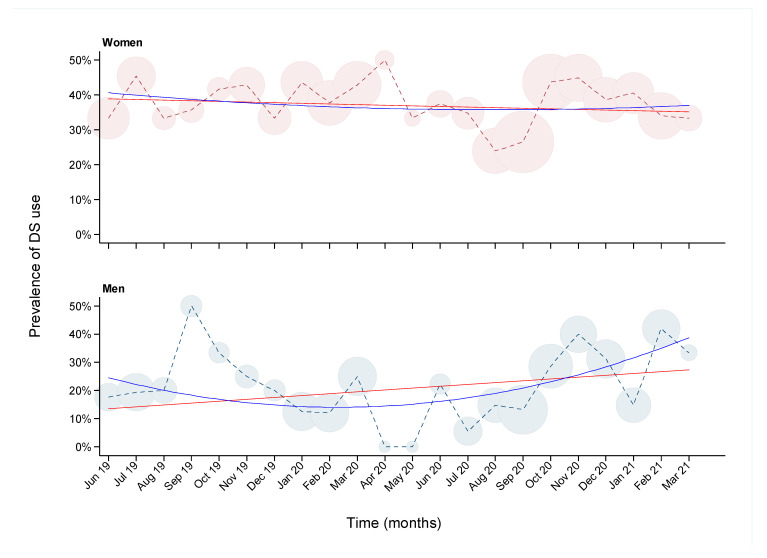
Prevalence of dietary supplement (DS) use over time among 748 women (top panel) and 489 men (bottom panel) participants of the Epirus Health Study. The X-axis represents months from June 2019 to March 2021. The Y-axis represents the percentage of DS use. Circles are proportional to the sample size at each month. Regression analyses with linear (red line) and quadratic (blue line) terms for month of participation are overlayed on the graphs.

The prevalence of DS use was 31.4% in the overall population but was higher in women than men (Table 1; 37.3% vs. 22.4%, *p*-value = 4.2^−08^). Among the women, 59.1% were pre-menopausal and 34.1% post-menopausal (not including women who had undergone hysterectomy or bilateral oophorectomy), but the prevalence of DS use did not differ significantly between the two groups (35.3% vs. 38.8% respectively; *p* value = 0.632). The standardized prevalence of DS use by age and sex, using the stratified Greek population as the standardization data, was 36.6% for women and 21.6% for men. Likewise, using the stratified Epirus population, the standardized prevalence was 36.2% for women and 21.2% for men. Figure 1 shows the prevalence of DS use during the recruitment period (June 2019 to March 2021) of the EHS in women and men. DS use varied widely, from 25% to 50% over the duration of the study in women, and from 0% to 50% in men. The prevalence showed a slight decreasing trend over time in women (linear regression beta: −0.16% per month; 95% confidence interval (CI): −0.08%, −0.23%), whereas in men it decreased during the first approximately 10 months and increased gradually after April 2020, showing an overall increasing trend (beta: 0.48% per month; 95% CI: 0.33%, 0.63%). We compared DS use before and after March 2020 to examine potential association with the COVID-19 pandemic. DS use in women did not differ significantly, adjusted for age (*p* value = 0.744), whereas in men a suggestive increase in DS use was found after the pandemic onset (*p* value = 0.08).

Table 2 shows the general characteristics of the study population according to DS use in women and men. Men DS users were more likely to be younger (*p*-value = 0.01), and women users slightly older (*p*-value = 0.06), compared to men and women nonusers, respectively. Men DS users were more physically active (*p*-value = 0.02), more likely to be employed (*p*-value = 0.02) and to take medications (*p*-value = 0.03) and had lower pulse wave velocity (*p*-value = 0.03) than men non-users. There was no statistically significant association between physical activity, employment status, medication use and pulse wave velocity and DS use in women. On the contrary, women using DS were more likely to report chronic diseases (*p* value < 0.01) and a worse general health status (*p* value = 0.03), had lower anthropometric indices (except for % body fat), lower diastolic blood pressure (DBP) and higher HDL cholesterol compared to women non-users (all *p* values ≤ 0.01), whereas no significant differences were observed for these variables in men.

When we used restricted cubic splines to examine the potential non-linear association between DS use and age (Supplementary Figures S1 and S2), the association was positive until middle-age and slightly negative afterwards in women. In men, the association did not deviate from linearity.

Table 3 and Table 4 report the results of multivariable-adjusted odds ratios (OR) of the independent correlates of DS use in the final model of the two-step approach in women and men, respectively. The odds of DS use were 70.5% higher (95% CI: 1.18–2.46) in women who reported a chronic disease or health condition. On the contrary, women who reported lost or removed teeth had 47.9% lower odds of DS use (95% CI: 0.35–0.78), and a 5 mmHg higher DBP was associated with a 16.5% lower odds of DS use (95% CI: 0.73–0.96). The association of age with DS use was non-linear (*p*-value = 0.04), as previously described. Borderline significant inverse associations were found in women for worse general health status and body mass index (BMI) (Table 3). Waist and hip circumference were excluded from the final model in women due to collinearity with BMI, but their exclusion did not change the other variables’ estimates. The results of the final model in women assessing the association between DS use and age linearly were similar (Supplementary Table S1).

In men, employment status was significantly associated with DS use, with employed men being 2.5 times more likely to use DS compared to unemployed/retired men (95% CI: 1.21–5.29) (Table 4). At a statistical significance level of 0.10, smoking cessation, increased MET hours/week, presence of chronic health conditions and medication use were positively associated with DS use, whereas an inverse association was found for BMI. The mean pulse wave velocity was removed from the final model because it showed collinearity with age.

The sensitivity analysis using the Collett’s procedure showed similar findings with the two-step approach for both women and men (data not shown).

## 4. Discussion

We investigated the prevalence of DS use and its determinants in 1237 participants of the Epirus Health Study cohort. The prevalence of DS use was substantially higher in women compared to men, and their correlates also differed by sex, although markers of both worse and good health were identified as correlated of DS use in both sexes. In women, age was positively associated with DS use until middle-age and slightly negatively afterwards; presence of a chronic health condition was also positively associated with DS use, whereas teeth loss and DBP were inversely associated. A positive association for general health status and an inverse association for BMI were borderline significant. In men, being employed was the only factor that was significantly associated with higher DS use, with BMI showing a suggestive negative association and smoking cessation, physical activity, presence of chronic disease or health condition and medication use showing suggestive positive associations.

This is the first contemporary study to describe the prevalence of DS use in a Greek adult population. The standardized prevalence of DS use by age and sex, using the stratified Greek population as the standardization data, was 36.6% for women and 21.6% for men. According to studies based on national surveys from other countries, the prevalence of DS use in adult populations among countries varied [7,9,26]. The highest prevalence rates were reported in a Danish national survey with 60% of women and 51% of men using DS [5], and in the US 2017–2018 NHANES survey, with the respective estimates being 63.8% for women and 50.8% for men [2], whereas the lowest prevalence was reported in a Chinese national survey where only 0.84% of women and 0.60% of men aged 6 years or above used DS [11]. Differences in population characteristics and in the definitions of DS across studies make difficult any attempts for comparisons between populations. However, regardless of the varying results across studies, results from previous studies agree that the prevalence of DS use is higher among women than men [12,24,27,28,29,30], in line with our findings.

Middle-aged women and younger men in our study were more likely to use DS, but the association in men did not retain significance in the multivariable model. Our findings regarding women are in agreement with the results of a 2012–2016 national Dutch survey, which showed that DS consumption for adult women increased with age until the age group of 51 to 70 years and decreased afterwards, whereas men 51 to 70 years had the lowest percentage of DS use compared to the other adult age groups [9]. Furthermore, in a Swiss population, DS use increased with age until the age group of 45–64 years and decreased afterwards, irrespectively of sex [24]. Positive linear trends have been reported in other studies [26,31,32].

We found that employed men were 2.5 times more likely to use DS compared to men of other employment statuses. A possible explanation is that employed men are more likely to have higher socio-economic and educational status, which has been linked to DS use [8,12,14,15]. The differing categorizations of employment status across the limited studies that have explored this association prohibit direct comparison with our results. In a French population, DS use was increased among men and women who were in service, compared to students, unemployed and retired individuals [27]. In an Italian sample, the highest percentage of DS users was among students, managers/professionals and employees [29]. In Japan, farmers and fishermen were the least likely occupation category to use DS [31]. No significant association between professional status and DS use was found in a Swiss study [24].

In the present study, participants who reported personal history of chronic diseases or health conditions were more likely to use DS (the association was significant for women and suggestive for men), in agreement with results from previous studies [33,34]. About 1 in 3 participants of a French cohort reported “solve or overcome health problems” as a reason for DS use [27]; likewise, “to recover from disease” was the second most frequently reported reason for using DS among Bangladeshi adults [35], while 26.2% of female participants in a Saudi Arabian study reported “injury or illness” as the main reason for DS use [36]. A suggestive association was also found between medication use and DS use among men. Similar results were reported in a 2015 population-based study in Dubai and in a Danish study [12,37], likewise in a study in a UK population; however, the results in the latter lost significance after adjustment for age and sex [30].

In contrast, we found a borderline significant association between self-assessed general health status and DS use in women, as those who reported very good health status were more likely to use DS compared to those who reported good health status or lower, although for the latter the data were limited. Results from previous research have been mixed. Participants of a large UK study who perceived their health as good or excellent were more likely to use DS than those reporting poor or fair health status [30]. In the 2011–2014 NHANES, 65.1% of women reporting excellent or very good health and 56.4% reporting good health were DS users, with a similar trend being reported for men [14]. Data from a 1996–1997 national representative Swedish sample showed that participants with subjective excellent health status used significantly less DS compared to those of other health statuses [38]. Results from other studies showed no significant association between self-perceived health status and DS use [26,29,39]. According to our study, women using DS were significantly more likely to have intact teeth. This agrees with results from previous studies suggesting that vitamin D supplementation may be associated with better periodontal health [40,41]. In a Swedish study, the frequency of dental check-ups was associated with increased DS use [38]. Good oral hygiene might be considered an indicator of health awareness and a healthy lifestyle, both related to DS consumption [7,15,27,32,42].

In the present study we found evidence for an inverse association between DBP and DS use in women. The potential lowering effect of DS use on blood pressure has a biological basis [43,44] and several ingredients used in DS have been linked to lower DBP [45,46,47]. In a recent meta-analysis of randomized controlled trials, Li et al. found a borderline significant inverse association between multivitamin–multimineral use and DBP, which was significant in obese individuals or those with chronic diseases [48]. Additionally, lower DBP has been associated with factors and behaviors indicative of a healthy lifestyle [49,50] which, as already mentioned, has been related to DS use. Furthermore, high blood pressure measurements and history of hypertension have been negatively associated with DS use [10,30].

Our finding that BMI was inversely associated with DS use for both women and men (borderline significantly and suggestively, respectively) is also consistent with results from previous studies [27]. Obese individuals have been associated with lower DS use compared to individuals with normal BMI [24,31,39,51]. Our data also revealed a suggestive positive association between physical activity and DS use in men. In agreement, DS use has been associated with physical activity in many previous studies, for both men and women [24,26,39,52]. We also found a suggestive positive association in men between DS use and former smoking compared to never smoking. Increased supplement use by former smokers might be an attempt to minimize the detrimental effects of their previous habit to their health. Findings from previous studies regarding smoking are mixed; some are similar to ours, irrespectively of sex [12,24]; others indicate that men who were former-, never-, or non-smokers were more likely to use DS than current smokers [26,31]; in a Danish national survey [5], Tetens et al. found no significant association.

In the present study we found a suggestive increase in DS use after the onset of the COVID-19 pandemic in men, but not in women. Several online surveys have been conducted regarding DS use during the pandemic. In a Greek survey, 19% of the participants reported that they started or increased the frequency of DS use during the pandemic [53]. In a Chinese survey conducted in March 2020, Zhao et al. found that 31.2% of the participants consumed DS or Chinese herbs to cope with COVID-19 [54]; similarly, in a survey conducted in Spain, at least 21.3% of the participants reported use of vitamins and mineral supplements, mostly women [55]. However, in a survey conducted in Hong Kong, no significant difference in DS use was found before vs. during the pandemic [56].

This study has several strengths. It is a population-based study conducted in Greece and has captured a wide range of potential correlates of DS use. Information was recorded through face-to-face interviews by trained interviewers. Anthropometric and clinical measurements were evaluated objectively by trained health professionals. Thorough regression modelling techniques were used to identify independent correlates of DS use. However, there are several limitations that also need to be considered. First, DS use was captured only at recruitment and may not reflect habitual supplement use. In addition, data about the type and dose of DS use were self-reported and incomplete. Finally, the cross-sectional study design precludes investigation of any causal relationships and the relatively small sample size did not allow us to make more detailed investigations in subgroups of the population by age and other characteristics.

In summary, a substantial prevalence of DS use was found in a non-random sample of the Greek population, especially in women. Different correlates of DS use by sex were identified, which should be replicated in independent and ideally representative studies, before they can be considered to inform public health policies. Since many studies have failed to show a clear benefit from taking supplements in the general population for prevention of major chronic diseases [57], similar research will aid the formulation of appropriate and effective interventions for the proper use of DS.

## Figures and Tables

**Table 1:** Baseline characteristics of the Epirus Health Study participants overall and according to sex.

	Total (*n* = 1237)	Women (*n* = 748)	Men (*n* = 489)	*p*-Value
*Demographics*				
Age (years)	48.1 (11.0)	48.2 (10.8)	48.0 (11.5)	0.79
Education (years)				
≤12	408 (33.0)	241 (32.3)	167 (34.2)	
>12	827 (67.0)	505 (67.7)	322 (65.8)	0.50
Employment status				
Employed	927 (75.3)	538 (72.3)	389 (79.9)	
Other	304 (24.7)	206 (27.7)	98 (20.1)	2.6^−03^
After-tax income (€/month)				
≤500	151 (13.1)	117 (16.8)	34 (7.5)	
501–900	298 (25.9)	205 (29.4)	93 (20.5)	
901–1400	515 (44.7)	306 (43.9)	209 (46.0)	
>1400	187 (16.2)	69 (9.9)	118 (26.0)	3.1^−15^
*Lifestyle*				
PSQI	4.9 (2.5)	5.1 (2.7)	4.5 (2.1)	2.7^−06^
MEDAS	7.2 (1.8)	7.4 (1.7)	7.1 (1.8)	3.2^−03^
Physical activity (MET-hours/week)	15.3 (20.1)	13.6 (18.8)	18.0 (21.7)	1.9^−04^
Smoking status				
Never smokers	423 (34.2)	278 (37.2)	145 (29.7)	
Former smokers	431 (34.9)	234 (31.3)	197 (40.3)	
Current smokers	382 (30.9)	235 (31.5)	147 (30.1)	2.6^−03^
Alcohol consumption				
Never	151 (12.2)	114 (15.3)	37 (7.6)	
Less than once/month	362 (29.3)	261 (34.9)	101 (20.7)	
1–3 times/month	211 (17.1)	125 (16.7)	86 (17.6)	
1–2 times/week	342 (27.7)	180 (24.1)	162 (33.1)	
At least 3 times/week	170 (13.8)	67 (9.0)	103 (21.1)	1.8^−15^
Cellphone use at least once/week (years)				
≤10	81 (6.6)	64 (8.6)	17 (3.5)	
11–15	149 (12.1)	101 (13.5)	48 (9.8)	
16–20	273 (22.1)	162 (21.7)	111 (22.7)	
>20	733 (59.3)	420 (56.2)	313 (64.0)	4.0^−04^
Dietary supplement use during last week	384 (31.4)	276 (37.3)	108 (22.4)	4.2^−08^
*Personal medical history (self-reported)*				
General health status				
Very good	460 (37.2)	256 (34.3)	204 (41.7)	
Good	626 (50.7)	390 (52.3)	236 (48.3)	
Moderate/Bad/Very bad	149 (12.1)	100 (13.4)	49 (10.0)	0.02
Chronic health condition	507 (41.2)	338 (45.4)	169 (34.8)	2.5^−04^
Limitation of usual activities during last year because of a health issue	191 (15.5)	124 (16.6)	67 (13.7)	0.17
Symptoms of stress during last 2 weeks	44 (3.6)	37 (5.0)	7 (1.4)	1.1^−03^
Symptoms of depression during last 2 weeks	38 (3.1)	31 (4.1)	7 (1.4)	0.01
Diabetes mellitus diagnosis	62 (5.0)	43 (5.8)	19 (3.9)	0.14
High cholesterol diagnosis	326 (26.5)	201 (26.9)	125 (25.7)	0.63
High blood pressure diagnosis	168 (13.6)	80 (10.7)	88 (18.0)	2.5^−04^
Bleeding gums	424 (34.3)	268 (35.9)	156 (31.9)	0.15
Lost/removed teeth (excl. wisdom teeth)	743 (61.5)	453 (62.2)	290 (60.3)	0.50
No. of dental fillings	3.3 (3.7)	3.4 (3.9)	3.2 (3.4)	0.55
Hearing impairment	92 (7.4)	48 (6.4)	44 (9.0)	0.09
Weight change since last year				
Same weight	650 (52.7)	363 (48.6)	287 (58.9)	
Weight gain	381 (30.9)	271 (36.3)	110 (22.6)	
Weight loss	203 (16.5)	113 (15.1)	90 (18.5)	2.4^−06^
Weight at 18 years (kg)	62.8 (12.5)	56.6 (8.6)	73.3 (10.9)	3.0^−131^
No. of drugs taken during last week				
0	777 (63.4)	451 (60.8)	326 (67.5)	
1	244 (19.9)	162 (21.8)	82 (17.0)	
≥2	204 (16.7)	129 (17.4)	75 (15.5)	0.05
*Clinical examinations*				
BMI (kg/m^2^)	26.5 (4.8)	25.7 (4.9)	27.7 (4.4)	2.6^−12^
Waist circumference (cm)	93.7 (13.8)	88.7 (12.6)	101.1 (12.1)	4.7^−58^
Hip circumference (cm)	101.9 (8.0)	102.0 (8.4)	101.6 (7.3)	0.35
Fat mass (%) ^1^	28.5 (7.7)	31.9 (6.9)	23.5 (5.9)	9.2^−89^
Fasting glucose (mg/dL)	86.3 (15.2)	84.3 (15.2)	89.4 (14.6)	9.3^−09^
Total cholesterol (mg/dL)	195.1 (34.2)	195.7 (33.6)	194.2 (35.3)	0.44
LDL cholesterol (mg/dL)	126.2 (34.0)	123.3 (33.8)	130.5 (33.9)	3.5^−04^
HDL cholesterol (mg/dL)	54.4 (12.4)	58.8 (12.1)	47.7 (9.5)	2.6^−57^
Triglycerides (mg/dL)	96.2 (57.7)	85.2 (50.3)	112.9 (63.8)	1.7^−16^
Systolic blood pressure (mmHg) ^2^	119.2 (13.4)	116.1 (13.1)	123.9 (12.5)	2.5^−23^
Diastolic blood pressure (mmHg) ^2^	77.6 (10.4)	75.1 (9.6)	81.5 (10.3)	1.4^−26^
Pulse wave velocity ^2^	6.9 (1.4)	6.8 (1.4)	7.1 (1.4)	1.1^−03^

Abbreviations: PSQI; Pittsburgh Sleep Quality Index, MEDAS; Mediterranean Diet Adherence Screener, MET; Metabolic Equivalent of Energy Expenditure, BMI; Body Mass Index, LDL; Low-density lipoprotein, HDL; High-density lipoprotein. Characteristics are presented as mean (standard deviation) for continuous variables and frequency (percentage) for categorical variables. Independent samples *t*-tests or chi-squared tests were used for comparing associations by sex. ^1^ Fat mass was measured using bioelectrical impedance analysis. ^2^ Systolic, diastolic blood pressure and pulse wave velocity were measured as average of three consecutive measurements with an arterial stiffness monitor.

**Table 2:** Baseline characteristics of the Epirus Health Study participants according to sex and dietary supplement (DS) use.

	Women (*n* = 740)			Men (*n* = 482)		
	No DS Users (*n* = 464)	DS Users (*n* = 276)	*p*-Value	No DS Users (*n* = 374)	DS Users (*n* = 108)	*p*-Value
*Demographics*						
Age (years)	47.5 (10.9)	49.1 (10.4)	0.06	48.6 (11.5)	45.5 (10.7)	0.01
Education (years)						
≤12	153 (33.0)	87 (31.5)		129 (34.5)	35 (32.4)	
>12	311 (67.0)	189 (68.5)	0.68	245 (65.5)	73 (67.6)	0.69
Employment status						
Employed	337 (72.9)	197 (71.1)		291 (78.0)	94 (87.9)	
Other	125 (27.1)	80 (28.9)	0.59	82 (22.0)	13 (12.1)	0.02
After-tax income (€/month)						
≤500	78 (18.0)	39 (15.1)		26 (7.4)	7 (7.2)	
501–900	139 (32.1)	65 (25.1)		68 (19.4)	24 (24.7)	
901–1400	174 (40.2)	129 (49.8)		160 (45.7)	45 (46.4)	
>1400	42 (9.7)	26 (10.0)	0.07	96 (27.4)	21 (21.6)	0.57
*Lifestyle*						
PSQI	5.1 (2.5)	5.2 (2.9)	0.54	4.5 (2.1)	4.2 (1.9)	0.31
MEDAS	7.4 (1.7)	7.4 (1.7)	0.84	7.1 (1.8)	7.1 (1.7)	0.70
Physical activity (MET-hours/week)	13.2 (19.9)	14.3 (16.9)	0.46	16.6 (20.9)	22.1 (22.4)	0.02
Smoking status						
Never smokers	177 (38.1)	98 (35.5)		116 (31.0)	28 (25.9)	
Former smokers	139 (30.0)	94 (34.1)		140 (37.4)	53 (49.1)	
Current smokers	148 (31.9)	84 (30.4)	0.50	118 (31.6)	27 (25.0)	0.09
Alcohol consumption						
Never	73 (15.7)	39 (14.1)		25 (6.7)	12 (11.1)	
Less than once/month	163 (35.1)	95 (34.4)		78 (20.9)	22 (20.4)	
1–3 times/month	78 (16.8)	46 (16.7)		67 (17.9)	18 (16.7)	
1–2 times/week	107 (23.1)	72 (26.1)		117 (31.3)	41 (38.0)	
At least 3 times/week	43 (9.3)	24 (8.7)	0.90	87 (23.3)	15 (13.9)	0.14
Cellphone use at least once/week (years)						
≤10	41 (8.8)	21 (7.6)		14 (3.7)	2 (1.9)	
11–15	61 (13.1)	40 (14.5)		35 (9.4)	12 (11.1)	
16–20	101 (21.8)	59 (21.4)		85 (22.7)	23 (21.3)	
>20	261 (56.3)	156 (56.5)	0.91	240 (64.2)	71 (65.7)	0.73
*Personal medical history (self-reported)*						
General health status						
Very good	176 (37.9)	79 (28.6)		158 (42.2)	45 (41.7)	
Good	228 (49.1)	158 (57.2)		179 (47.9)	53 (49.1)	
Moderate/bad/very bad	60 (12.9)	39 (14.1)	0.03	37 (9.9)	10 (9.3)	0.97
Chronic health condition	188 (40.7)	145 (52.5)	1.8^−03^	121 (32.6)	45 (42.1)	0.07
Limitation of usual activities during last year because of a health issue	69 (14.9)	53 (19.2)	0.13	51 (13.6)	15 (13.9)	0.95
Symptoms of stress during last 2 weeks	26 (5.6)	11 (4.0)	0.33	4 (1.1)	2 (1.9)	0.52
Symptoms of depression during last 2 weeks	17 (3.7)	13 (4.7)	0.49	5 (1.3)	1 (0.9)	0.73
Diabetes mellitus diagnosis	28 (6.0)	14 (5.1)	0.58	17 (4.5)	1 (0.9)	0.08
High cholesterol diagnosis	119 (25.6)	78 (28.3)	0.44	91 (24.5)	30 (28.0)	0.45
High blood pressure diagnosis	55 (11.9)	25 (9.1)	0.24	69 (18.5)	15 (13.9)	0.27
Bleeding gums	169 (36.4)	99 (35.9)	0.88	118 (31.6)	35 (32.4)	0.87
Lost/removed teeth (excl. wisdom teeth)	292 (64.6)	157 (58.1)	0.08	228 (61.8)	58 (54.7)	0.19
No. of dental fillings	3.3 (3.9)	3.4 (3.8)	0.66	3.3 (3.5)	2.9 (2.9)	0.25
Hearing impairment	25 (5.4)	22 (8.0)	0.16	35 (9.4)	8 (7.4)	0.53
Weight change since last year						
Same weight	233 (50.2)	128 (46.4)		223 (59.8)	59 (55.1)	
Weight gain	166 (35.8)	102 (37.0)		79 (21.2)	30 (28.0)	
Weight loss	65 (14.0)	46 (16.7)	0.50	71 (19.0)	18 (16.8)	0.33
Weight at 18 years (kg)	56.9 (9.0)	56.1 (8.1)	0.22	72.8 (10.8)	74.1 (10.6)	0.31
No. of drugs taken during last week						
0	292 (63.3)	159 (57.6)		261 (70.5)	64 (59.3)	
1	96 (20.8)	64 (23.2)		54 (14.6)	27 (25.0)	
≥2	73 (15.8)	53 (19.2)	0.28	55 (14.9)	17 (15.7)	0.03
*Clinical examinations*						
BMI (kg/m^2^)	26.1 (5.2)	25.0 (4.3)	0.01	27.8 (4.4)	27.0 (4.1)	0.10
Waist circumference (cm)	89.5 (13.1)	87.1 (11.7)	0.01	101.5 (12.4)	99.8 (10.8)	0.21
Hip circumference (cm)	102.7 (8.6)	101.0 (8.1)	0.01	101.8 (7.4)	100.9 (7.2)	0.24
Fat mass (%) ^1^	32.0 (7.0)	31.6 (6.6)	0.51	23.7 (5.9)	22.7 (5.8)	0.16
Fasting glucose (mg/dL)	84.9 (17.9)	83.2 (9.2)	0.16	89.6 (15.7)	88.6 (9.5)	0.52
Total cholesterol (mg/dL)	195.7 (34.7)	195.6 (31.8)	0.99	196.0 (35.8)	189 (32.4)	0.07
LDL cholesterol (mg/dL)	124.0 (35.4)	121.8 (31.3)	0.41	131.8 (34.7)	126.8 (29.8)	0.19
HDL cholesterol (mg/dL)	58.0 (11.7)	60.4 (12.5)	0.01	48.0 (9.6)	47.0 (8.9)	0.34
Triglycerides (mg/dL)	87.6 (57.0)	80.2 (35.4)	0.06	113.2 (66.6)	111.3 (54.1)	0.79
Systolic blood pressure (mmHg) ^2^	116.8 (13.5)	115.0 (12.5)	0.08	123.9 (12.6)	123.2 (11.4)	0.63
Diastolic blood pressure (mmHg) ^2^	75.9 (9.9)	73.7 (8.7)	2.9^−03^	82.0 (10.2)	79.9 (10.4)	0.07
Pulse wave velocity ^2^	6.7 (1.4)	6.9 (1.3)	0.24	7.1 (1.4)	6.8 (1.2)	0.03

Abbreviations: PSQI; Pittsburgh Sleep Quality Index, MEDAS; Mediterranean Diet Adherence Screener, MET; Metabolic Equivalent of Energy Expenditure, BMI; Body Mass Index, LDL; Low-density lipoprotein, HDL; High-density lipoprotein. Characteristics are presented as mean (standard deviation) for continuous variables and frequency (percentage) for categorical variables. Independent samples *t*-tests or chi-squared tests were used for comparing associations by DS use. ^1^ Fat mass was measured using bioelectrical impedance analysis. ^2^ Systolic, diastolic blood pressure and pulse wave velocity were measured as average of three consecutive measurements with an arterial stiffness monitor.

**Table 3:** Adjusted odds ratios (OR) and 95% confidence intervals (CI) for the association between dietary supplement use and demographic characteristics, lifestyle factors and clinical examinations among 641 women participants of the Epirus Health Study; the association between DS use and age was assessed non-linearly.

Variable	OR (95% CI)	*p*-Value	Overall *p*-Value ^1^
Age	N/A ^2^	N/A ^2^	0.04
After-tax income (€/month)			
≤500	Reference		
501–900	0.900 (0.527–1.539)	0.70	
901–1400	1.254 (0.752–2.090)	0.39	
>1400	1.045 (0.521–2.095)	0.90	0.46
General health status			
Very good	Reference		
Good	0.562 (0.309–1.023)	0.06	
Moderate/bad/very bad	0.896 (0.526–1.527)	0.69	0.05
Chronic health condition			
No	Reference		
Yes	1.705 (1.182–2.458)	4.3^−03^	
Lost/removed teeth (excl. wisdom teeth)			
No	Reference		
Yes	0.521 (0.351–0.775)	1.3^−03^	
BMI (per 5 kg/m^2^)	0.793 (0.631–0.997)	0.05	
HDL cholesterol (per 5 mg/dL)	1.032 (0.953–1.119)	0.43	
Triglycerides (per 5 mg/dL)	0.992 (0.968–1.017)	0.54	
Systolic blood pressure (per 5 mmHg) ^3^	1.043 (0.937–1.162)	0.44	
Diastolic blood pressure (per 5 mmHg) ^3^	0.835 (0.725–0.961)	0.01	

Abbreviations: BMI; Body Mass Index, HDL; High-density lipoprotein. All variables associated univariably with DS use at the 10% statistical significance threshold from Table 2 were entered in a multivariable model. Among the anthropometric variables, only BMI was left in the model to avoid multi-collinearity. ^1^ Overall *p*-value was calculated by overall Wald test. ^2^ Age was included in the model using restricted cubic splines with five knots. ^3^ Systolic and diastolic blood pressure were measured as average of three consecutive measurements with an arterial stiffness monitor.

**Table 4:** Adjusted odds ratios (OR) and 95% confidence intervals (CI) for the association between dietary supplement use and demographic characteristics, lifestyle factors and clinical examinations among 452 men participants of the Epirus Health Study.

Variable	OR (95% CI)	*p*-Value	Overall *p*-Value ^1^
Age (per 5 years)	0.914 (0.804–1.038)	0.16	
Employment status			
Other	Reference		
Employed	2.526 (1.208–5.285)	0.01	
Smoking status			
Never smokers	Reference		
Former smokers	1.634 (0.931–2.867)	0.09	
Current smokers	0.874 (0.462–1.652)	0.68	0.07
Physical activity (per 5 MET-hours/week)	1.046 (0.993–1.101)	0.09	
Chronic health condition			
No	Reference		
Yes	1.661 (0.935–2.949)	0.08	
No. of drugs taken during last week			
0	Reference		
1	2.138 (1.102–4.150)	0.03	
≥2	1.268 (0.542–2.964)	0.58	0.07
BMI (per 5 kg/m^2^)	0.739 (0.540–1.013)	0.06	
Total cholesterol (per 5 mg/dL)	0.987 (0.952–1.024)	0.49	
Diastolic blood pressure (per 5 mmHg) ^2^	0.973 (0.856–1.105)	0.67	

Abbreviations: MET; Metabolic Equivalent of Energy Expenditure, BMI; Body Mass Index. All variables associated univariably with DS use at the 10% statistical significance threshold from Table 2 were entered in a multivariable model. Pulse wave velocity was removed from the model to avoid multi-collinearity with age. ^1^ Overall *p*-value was calculated by overall Wald test. ^2^ Diastolic blood pressure was measured as average of three consecutive measurements with an arterial stiffness monitor.

## Supplementary Materials

The following are available online at https://www.mdpi.com/article/10.3390/nu13082857/s1, Figure S1: Probability of DS use vs. age in women, Figure S2: Probability of DS use vs. age in men, Table S1: Adjusted odds ratios (OR) and 95% confidence intervals (CI) for the association between dietary supplement use and demographic characteristics, lifestyle factors and clinical examinations among 641 women participants of the Epirus Health Study; the association between DS use and age was assessed linearly.

## References

[1] Dietary Supplements Market Size & Trends Report, 2021–2028. https://www.grandviewresearch.com/industry-analysis/dietary-supplements-market.

[2] Mishra S., Stierman B., Gahche J.J., Potischman N. (2021). Dietary Supplement Use among Adults: United States, 2017–2018. NHCS Data Brief No 399.

[3] Briefel R.R., Johnson C.L. (2004). Secular trends in dietary intake in the United States. Annu. Rev. Nutr..

[4] Messerer M., Johansson S.E., Wolk A. (2001). Use of dietary supplements and natural remedies increased dramatically during the 1990s. J. Intern. Med..

[5] Tetens I., Biltoft-Jensen A., Spagner C., Christensen T., Gille M., Bügel S., Rasmussen L. (2011). Intake of micronutrients among Danish adult users and non-users of dietary supplements. Food Nutr. Res..

[6] Use of Nutritional Supplements in Canada 2015. https://www150.statcan.gc.ca/n1/pub/82-625-x/2017001/article/14831-eng.htm.

[7] Kang M., Kim D.W., Baek Y.J., Moon S.-H., Jung H.J., Song Y.J., Paik H.-Y. (2014). Dietary supplement use and its effect on nutrient intake in Korean adult population in the Korea National Health and Nutrition Examination Survey IV (2007–2009) data. Eur. J. Clin. Nutr..

[8] Guo X., Willows N., Kuhle S., Jhangri G., Veugelers P.J. (2009). Use of vitamin and mineral supplements among Canadian adults. Can. J. Public Health.

[9] van Rossum C.T.M., Buurma-Rethans E.J.M., Dinnissen C.S., Beukers M.H., Brants H.A.M., Dekkers A.L.M., Ocké M.C. (2020). The Diet of the Dutch—Results of the Dutch National Food Consumption Survey 2012–2016.

[10] Burnett A.J., Livingstone K.M., Woods J.L., McNaughton S.A. (2017). Dietary supplement use among Australian adults: Findings from the 2011–2012 national nutrition and physical activity survey. Nutrients.

[11] Gong W., Liu A., Yao Y., Ma Y., Ding C., Song C., Yuan F., Zhang Y., Feng G., Chen Z. (2018). Nutrient Supplement Use among the Chinese Population: A Cross-Sectional Study of the 2010–2012 China Nutrition and Health Surveillance. Nutrients.

[12] Abdulla N.M., Aziz F., Blair I., Grivna M., Adam B., Loney T. (2019). Prevalence of, and factors associated with health supplement use in Dubai, United Arab Emirates: A population-based cross-sectional study. BMC Complement. Altern. Med..

[13] Sousa A.G., Costa T.H.M.D. (2021). Diet and supplement assessment in a Brazilian urban population. Rev. Saúde Pública.

[14] Cowan A.E., Jun S., Gahche J.J., Tooze J.A., Dwyer J.T., Eicher-Miller H.A., Bhadra A., Guenther P.M., Potischman N., Dodd K.W. (2018). Dietary supplement use differs by socioeconomic and health-related characteristics among U.S. adults, NHANES 2011–2014. Nutrients.

[15] Reinert A., Rohrmann S., Becker N., Linseisen J. (2007). Lifestyle and diet in people using dietary supplements: A German cohort study. Eur. J. Nutr..

[16] Skeie G., Braaten T., Hjartåker A., Lentjes M., Amiano P., Jakszyn P., Pala V., Palanca A., Niekerk E.M., Verhagen H. (2009). Use of dietary supplements in the european prospective investigation into cancer and nutrition calibration study (EPIC). Eur. J. Clin. Nutr..

[17] Lidell E., Luepker R., Baigi A., Lagiou A., Hildingh C. (2008). Medication usage among young adult women: A comparison between Sweden, the USA, and Greece. Nurs. Health Sci..

[18] The EQUATOR Network The Strengthening the Reporting of Observational Studies in Epidemiology (STROBE) Statement: Guidelines for Reporting Observational Studies. https://www.equator-network.org/reporting-guidelines/strobe/.

[19] Kanellopoulou A., Koskeridis F., Markozannes G., Bouras E., Soutziou C., Chaliasos K., Doumas M.T., Sigounas D.E., Tzovaras V.T., Panos A. (2021). Awareness, knowledge and trust in the Greek authorities towards COVID-19 pandemic: Results from the Epirus Health Study cohort. BMC Public Health.

[20] EUR-Lex EUR-Lex—32002L0046—EN. https://eur-lex.europa.eu/legal-content/EN/TXT/?uri=CELEX:32002L0046.

[21] Martínez-González M.A., García-Arellano A., Toledo E., Salas-Salvadó J., Buil-Cosiales P., Corella D., Covas M.I., Schröder H., Arós F., Gómez-Gracia E. (2012). A 14-item mediterranean diet assessment tool and obesity indexes among high-risk subjects: The PREDIMED trial. PLoS ONE.

[22] Buysse D.J., Reynolds C.F., Monk T.H., Berman S.R., Kupfer D.J. (1989). The Pittsburgh sleep quality index: A new instrument for psychiatric practice and research. Psychiatry Res..

[23] Ainsworth B.E., Haskell W.L., Leon A.S., Jacobs D.R., Montoye H.J., Sallis J.F., Paffenbarger R.S. (1993). Compendium of physical activities: Classification of energy costs of human physical activities. Med. Sci. Sports Exerc..

[24] Marques-Vidal P., Pécoud A., Hayoz D., Paccaud F., Mooser V., Waeber G., Vollenweider P. (2009). Prevalence and characteristics of vitamin or dietary supplement users in Lausanne, Switzerland: The CoLaus study. Eur. J. Clin. Nutr..

[25] Collett D. (2015). Chapter 3.6.1 Variable selection procedures. Modelling Survival Data in Medical Research.

[26] O’brien S.K., Malacova E., Sherriff J.L., Black L.J. (2017). The Prevalence and Predictors of Dietary Supplement Use in the Australian Population. Nutrients.

[27] Pouchieu C., Andreeva V.A., Péneau S., Kesse-Guyot E., Lassale C., Hercberg S., Touvier M. (2013). Sociodemographic, lifestyle and dietary correlates of dietary supplement use in a large sample of French adults: Results from the NutriNet-Santé cohort study. Br. J. Nutr..

[28] Li K., Kaaks R., Linseisen J., Rohrmann S. (2012). Vitamin/mineral supplementation and cancer, cardiovascular, and all-cause mortality in a German prospective cohort (EPIC-heidelberg). Eur. J. Nutr..

[29] Giammarioli S., Boniglia C., Carratù B., Ciarrocchi M., Chiarotti F., Mosca M., Sanzini E. (2013). Use of food supplements and determinants of usage in a sample Italian adult population. Public Health Nutr..

[30] Harrison R.A., Holt D., Pattison D.J., Elton P.J. (2004). Are those in need taking dietary supplements? A survey of 21 923 adults. Br. J. Nutr..

[31] Ishihara J., Sobue T., Yamamoto S., Sasaki S., Tsugane S. (2003). Demographics, lifestyles, health characteristics, and dietary intake among dietary supplement users in Japan. Int. J. Epidemiol..

[32] Kofoed C.L.F., Christensen J., Dragsted L.O., Tjønneland A., Roswall N. (2015). Determinants of dietary supplement use—Healthy individuals use dietary supplements. Br. J. Nutr..

[33] Jasti S., Siega-Riz A.M., Bentley M.E. (2003). Dietary Supplement Use in the Context of Health Disparities: Cultural, Ethnic and Demographic Determinants of Use. J. Nutr..

[34] He Y.N., Yang Z., Xu J., Sha Y.M., Ren Z.Y., Pang X.H., Zeng G., Zhai F.Y. (2008). Analysis on influence factors of dietary supplement used in population aged above 45 years in Beijing. Chin. J. Prev. Med..

[35] Dietary Supplements Use and Associated Determinants among Adult Population in Southern Bangladesh. https://www.researchgate.net/publication/327666000_Dietary_Supplements_Use_and_Associated_Determinants_Among_Adult_Population_in_Southern_Bangladesh.

[36] Alfawaz H., Khan N., Alfaifi A., Shahrani F.M., Al Tameem H.M., Al Otaibi S.F., Abudigin W.I., Al-Shayaa M.S., Al-Ghanim S.A., Al-Daghri N.M. (2017). Prevalence of dietary supplement use and associated factors among female college students in Saudi Arabia. BMC Women’s Health.

[37] Knudsen V.K., Rasmussen L.B., Haraldsdóttir J., Ovesen L., Bülow I., Knudsen N., Jørgensen T., Laurberg P., Perrild H. (2002). Use of dietary supplements in Denmark is associated with health and former smoking. Public Health Nutr..

[38] Messerer M., Johansson S.E., Wolk A. (2001). Sociodemographic and health behaviour factors among dietary supplement and natural remedy users. Eur. J. Clin. Nutr..

[39] Radimer K., Bindewald B., Hughes J., Ervin B., Swanson C., Picciano M.F. (2004). Dietary Supplement Use by US Adults: Data from the National Health and Nutrition Examination Survey. Am. J. Epidemiol..

[40] Miley D.D., Garcia M.N., Hildebolt C.F., Shannon W.D., Couture R.A., Anderson Spearie C.L., Dixon D.A., Langenwalter E.M., Mueller C., Civitelli R. (2009). Cross-Sectional Study of Vitamin D and Calcium Supplementation Effects on Chronic Periodontitis. J. Periodontol..

[41] Garcia M.N., Hildebolt C.F., Miley D.D., Dixon D.A., Couture R.A., Anderson Spearie C.L., Langenwalter E.M., Shannon W.D., Deych E., Mueller C. (2011). One-Year Effects of Vitamin D and Calcium Supplementation on Chronic Periodontitis. J. Periodontol..

[42] Kirk S.F., Cade J.E., Barrett J.H., Conner M. Diet and Lifestyle Characteristics Associated with Dietary Supplement Use in Women. https://www.cambridge.org/core.

[43] Tare M., Emmett S.J., Coleman H.A., Skordilis C., Eyles D.W., Morley R., Parkington H.C. (2011). Vitamin D insufficiency is associated with impaired vascular endothelial and smooth muscle function and hypertension in young rats. J. Physiol..

[44] Dakshinamurti K., Paulose C.S., Viswanathan M. (1990). Vitamin B6 and Hypertension. Ann. N. Y. Acad. Sci..

[45] Ried K., Fakler P., Stocks N.P. (2017). Effect of cocoa on blood pressure. Cochrane Database Syst. Rev..

[46] Miller P.E., van Elswyk M., Alexander D.D. (2014). Long-chain Omega-3 fatty acids eicosapentaenoic acid and docosahexaenoic acid and blood pressure: A meta-analysis of randomized controlled trials. Am. J. Hypertens..

[47] Dong J.Y., Qin L.Q., Zhang Z., Zhao Y., Wang J., Arigoni F., Zhang W. (2011). Effect of oral l-arginine supplementation on blood pressure: A meta-analysis of randomized, double-blind, placebo-controlled trials. Am. Heart J..

[48] Li K., Liu C., Kuang X., Deng Q., Zhao F., Li D. (2018). Effects of multivitamin and multimineral supplementation on blood pressure: A meta-analysis of 12 randomized controlled trials. Nutrients.

[49] Ndanuko R.N., Tapsell L.C., Charlton K.E., Neale E.P., Batterham M.J. (2016). Dietary Patterns and Blood Pressure in Adults: A Systematic Review and Meta-Analysis of Randomized Controlled Trials. Adv. Nutr..

[50] Landi F., Calvani R., Picca A., Tosato M., Martone A.M., Ortolani E., Sisto A., D’Angelo E., Serafini E., Desideri G. (2018). Body Mass Index is Strongly Associated with Hypertension: Results from the Longevity Check-Up 7+ Study. Nutrients.

[51] Foote J.A., Murphy S.P., Wilkens L.R., Hankin J.H., Henderson B.E., Kolonel L.N. (2003). Factors Associated with Dietary Supplement Use among Healthy Adults of Five Ethnicities. Am. J. Epidemiol..

[52] McNaughton S.A., Mishra G.D., Paul A.A., Prynne C.J., Wadsworth M.E. (2005). Supplement use is associated with health status and health-related behaviors in the 1946 British Birth Cohort. J. Nutr..

[53] Panagiotakos D., Kosti R.I., Pitsavos C. (2021). How will the way we live look different in the wake of the COVID-19 pandemic? A nutrition survey in Greece. Nutr. Health.

[54] Zhao A., Li Z., Ke Y., Huo S., Ma Y., Zhang Y., Zhang J., Ren Z. (2020). Dietary Diversity among Chinese Residents during the COVID-19 Outbreak and Its Associated Factors. Nutrients.

[55] Pérez-Rodrigo C., Citores M.G., Hervás Bárbara G., Ruiz-Litago F., Casis Sáenz L., Arija V., López-Sobaler A.M., Martínez de Victoria E., Ortega R.M., Partearroyo T. (2021). Patterns of Change in Dietary Habits and Physical Activity during Lockdown in Spain Due to the COVID-19 Pandemic. Nutrients.

[56] Lam C.S., Koon H.K., Chung V.C.H., Cheung Y.T. (2021). A public survey of traditional, complementary and integrative medicine use during the COVID-19 outbreak in Hong Kong. PLoS ONE.

[57] Moyer V.A. (2014). Vitamin, mineral, and multivitamin supplements for the primary prevention of cardiovascular disease and cancer: U.S. Preventive services Task Force recommendation statement. Ann. Intern. Med..

