# Supplementary material for: Prevalence and Determinants of Sex-Specific Dietary Supplement Use in a Greek Cohort
Source: Nutrients. 2021 Aug 20;13(8):2857. doi: 10.3390/nu13082857 (PMC8399686; doi:10.3390/nu13082857)
Supplement: Supplementary file 1 [file nutrients-13-02857-s001.zip › nutrient-1319782-supplement.pdf]

Table S1: Adjusted odds ratios (OR) and 95% confidence intervals (CI) for the association between dietary supplement use and demographic characteristics, lifestyle factors and clinical examinations among 641 female participants of the Epirus Health Study, with age assessed linearly

**Variable**	**OR (95% CI)**	***P-value***	***Overall p-value*[1]**
Age (per 5 years)	1.139 (1.029-1.261)	0.01	
After-tax income (€/month)			
≤500	Reference		
501-900	0.912 (0.536-1.551)	0.73	
901-1400	1.392 (0.846-2.292)	0.19	
>1400	1.163 (0.587-2.306)	0.66	0.20
General health status			
Very good	Reference		
Good	0.589 (0.325-1.066)	0.08	
Moderate/bad/very bad	0.921 (0.543-1.564)	0.76	0.06
Chronic health condition			
No	Reference		
Yes	1.672 (1.162-2.406)	5.7^{-03}	
Lost/removed teeth (excl. wisdom teeth)			
No	Reference		
Yes	0.548 (0.371-0.811)	2.6^{-03}	
BMI (per 5 kg/m^2)	0.818 (0.653-1.024)	0.08	
HDL cholesterol (per 5 mg/dL)	1.043 (0.964-1.129)	0.30	
Triglycerides (per 5 mg/dL)	0.993 (0.969- 1.018)	0.60	
Systolic blood pressure (per 5mmHg)[2]	1.026 (0.924-1.140)	0.63	
Diastolic blood pressure (per 5 mmHg)[2]	0.854 (0.745-0.980)	0.02	

All variables associated univariably with DS use at the 10% statistical significance threshold from Table 2 were entered in a multivariable model. Among the anthropometric variables, only BMI was left in the model to avoid multi-collinearity.

[1] Overall p-value was calculated by overall Wald test

[2] Systolic and diastolic blood pressure were measured as average of three consecutive measurements with an arterial stiffness monitor.

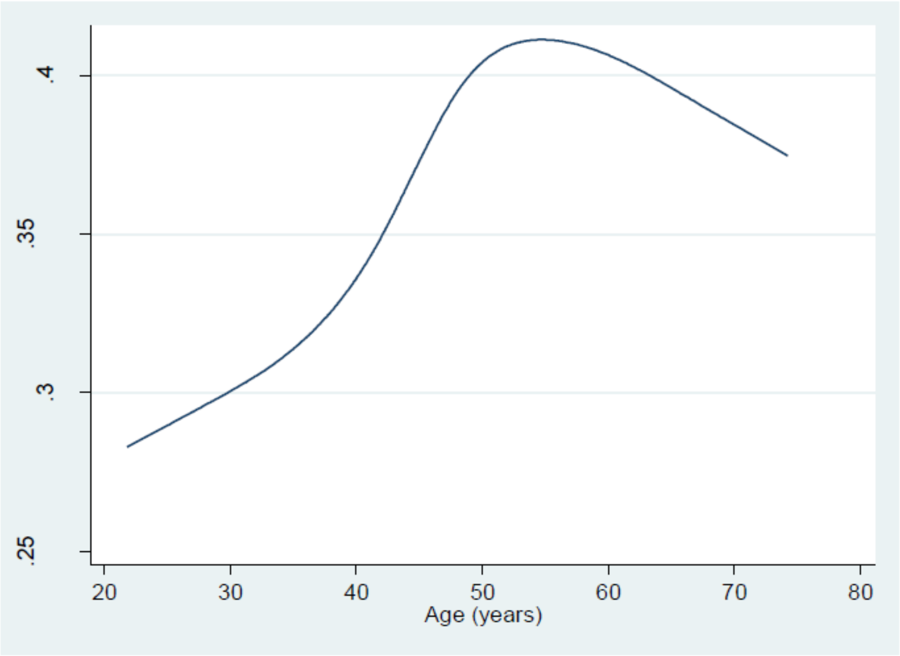

Figure S1: Probability of DS use vs. age in women.*

*The analysis was performed using restricted cubic splines with five knots.

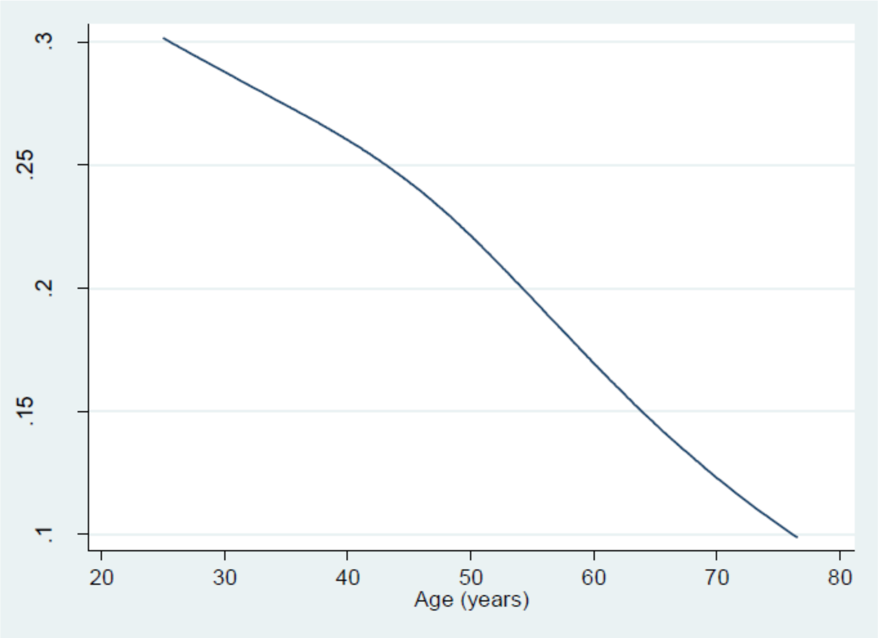

Figure S2: Probability of DS use vs. age in men.*

*The analysis was performed using restricted cubic splines with five knots.
